# Recent Advances and Perspectives in Lithium−Sulfur Pouch Cells

**DOI:** 10.3390/molecules26216341

**Published:** 2021-10-20

**Authors:** Weifeng Zhang, Shulian Li, Aijun Zhou, Huiyu Song, Zhiming Cui, Li Du

**Affiliations:** 1 The Key Laboratory of Fuel Cell Technology of Guangdong Province, School of Chemistry and Chemical Engineering, South China University of Technology, Guangzhou 510641, China; 201820121360@mail.scut.edu.cn (W.Z.); 201921023506@mail.scut.edu.cn (S.L.); hysong@scut.edu.cn (H.S.); zmcui@scut.edu.cn (Z.C.); 2 Yangtze Delta Region Institute (Huzhou), University of Electronic Science and Technology of China, Huzhou 313002, China; zhouaj@uestc.edu.cn

**Keywords:** lithium–sulfur batteries, pouch cells, lithium anode, polysulfide shuttling

## Abstract

Lithium–sulfur batteries (LSBs) are considered one of the most promising candidates for next-generation energy storage owing to their large energy capacity. Tremendous effort has been devoted to overcoming the inherent problems of LSBs to facilitate their commercialization, such as polysulfide shuttling and dendritic lithium growth. Pouch cells present additional challenges for LSBs as they require greater electrode active material utilization, a lower electrolyte–sulfur ratio, and more mechanically robust electrode architectures to ensure long-term cycling stability. In this review, the critical challenges facing practical Li–S pouch cells that dictate their energy density and long-term cyclability are summarized. Strategies and perspectives for every major pouch cell component—cathode/anode active materials and electrode construction, separator design, and electrolyte—are discussed with emphasis placed on approaches aimed at improving the reversible electrochemical conversion of sulfur and lithium anode protection for high-energy Li–S pouch cells.

## 1. Introduction

Commercial Li-ion batteries with an oxide intercalation cathode, a liquid organic electrolyte, a polymer separator, and a carbon-based anode have been widely used and developed in recent years. It has been generally accepted that next-generation secondary batteries must have an improved energy density and a minimized volume/weight ratio to promote the development of portable electronic devices and electrical vehicles that can operate longer on a single charge [1,2,3,4,5]. Among the many secondary battery chemistries proposed to take the reins from Li-ion batteries, lithium–sulfur batteries (LSBs) are one of the most promising candidates owing to their high theoretical specific capacity (1675 mAh g^−1^), high energy density (2500 Wh kg^−1^), low cost, and nontoxicity [6,7,8]. These attributes have made LSBs one of the most investigated systems in academic and industrial research laboratories. Standard LSBs operate on the electrochemical redox conversion of elemental sulfur, which delivers an ultrahigh theoretical energy density. Elemental sulfur combines with Li ions during discharge and is reduced to long-chain polysulfides (Li_2_S_x_ (4 ≤ x ≤ 8)) before ultimately being converted into short-chain Li_2_S_2_/Li_2_S at the end of the discharge process. However, LSBs cycled under practical conditions exhibit energy density and cycling stability far below theoretical expectations.

**Figure 1:**
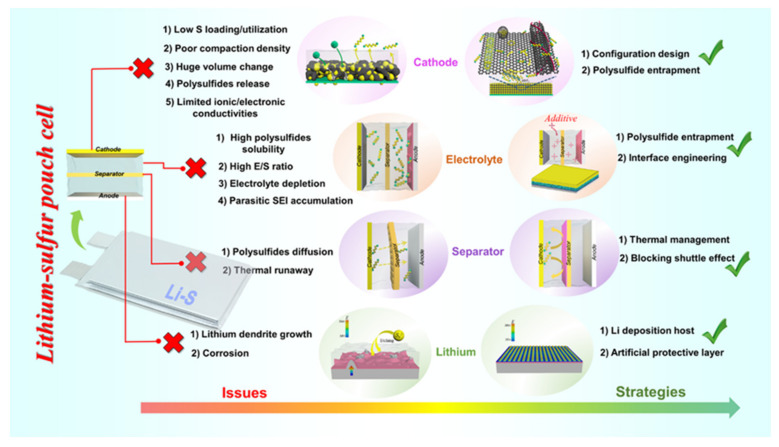
Overview of key issues and strategic solutions for the development of practical Li–S pouch cells [6,11,17,18].

Li–S cells have many intrinsic limitations that have been elaborately investigated and reviewed in previous literature [9,10]. Specifically, the energy density and cyclability of Li–S coin cells are limited by (1) loading and utilization of sulfur active material in the cathode composite, (2) polysulfide dissolution and shuttling in liquid electrolytes, (3) electrolyte depletion and interfacial degradation, and (4) dendritic lithium growth and the evolution of “dead Li”. Furthermore, the gravimetric and volumetric energy densities of Li–S cells explored in the research community are restricted by excessive usage of electrolyte and lithium metal during experimentation. Studies on these issues bring new understanding to the drawbacks of scaling small-scale cells used in research laboratories to large industrial-scale Li–S pouch cells [11,12]. Many of the aforementioned difficulties of the Li–S system are exacerbated when moving to lean electrolyte (low E/S ratio) and limited Li anode reservoir conditions; Li–S pouch cells cycled under these protocols suffer from much faster capacity degradation (Table 1). Additionally, the gravimetric energy density and cycling stability of large-scale Li–S pouch cells are significantly affected by sulfur active material mass loading, volumetric cell expansion, electrolyte depletion, thermal release, and the Li metal anode during practical operation (Figure 1) [7].

Optimization of Li–S battery performance under low E/S ratio conditions is typically performed with coin cells or small pouch cells. A plethora of strategies are being pursued to develop larger Li–S pouch cells that can satisfy all of the target parameters for commercialization—70% loading and 80% utilization of sulfur active material in cathode composite, an E/S ratio of 3 μL mg^−1^, 3-fold lithium excess, and long-term stability [19,20,21,22]. The bulk of these approaches related to sulfur cathode construction (host design, compaction density, thickness), electrolyte (type, additives, amount), separator design (thermal management, functionalization), and engineering the Li metal anode (deposition homogenization, protection) are summarized in Figure 1. In this work, we review each of these concepts and evaluate their validity in addressing the intrinsic problem in the Li–S pouch cell system operating under realistic conditions that they were designed to resolve.

## 2. Li–S Pouch Cell Cathodes

**Figure 2:**
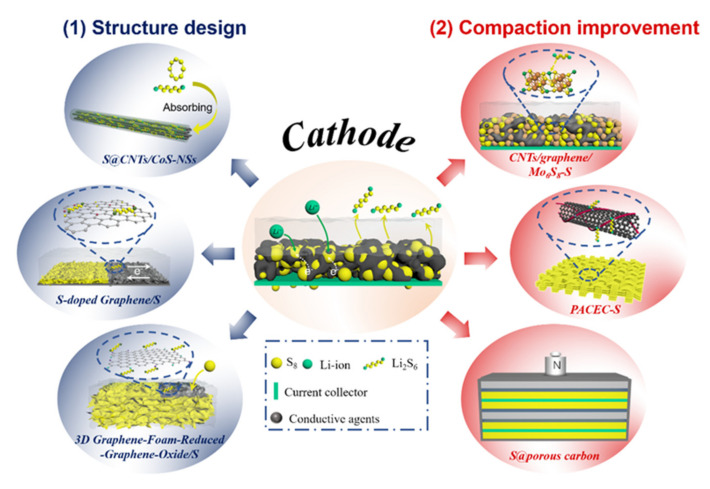
Composite cathode issues in Li–S batteries include low S loading/utilization, large volume change during cycling, poor compaction density, polysulfide dissolution, and limited electronic/ionic conductivities. Corresponding strategies for enhancing cathode composite architecture to improve electrochemical performance include (**1**) decorating nanosized sulfur in a robust hierarchical conductive matrix and (**2**) improving compaction with polar radical compounds, functional binders, and external stacking pressure.

Sulfur and the final discharge product of the Li–S battery, Li_2_S, are both electronic insulators. This property of the cathode active materials is one of the primary reasons for capacity degradation in Li–S batteries upon extended cycling (Figure 2). Additionally, the sulfur active material experiences a volume change of about 80% during conversion to Li_2_S, resulting in the physical contact loss between the active material and the electronically conductive additives in the cathode composite [23]. Poor compaction and electrolyte swelling in the cathode also trigger active material loss and electrolyte consumption. The precipitation of sulfur species causes compression strain–stress of the slurry during coating, which requires a cathode composite of varying thickness to overcome. Therefore, strategies to enhance sulfur loading and utilization and compaction of the cathode are crucial objectives in accelerating the industrial realization of Li–S pouch cells. Section 2.1 details advanced strategies focused on building robust hierarchical conductive hosts for a cathode composite with high S loading, limited volume change, and low electrolyte consumption [24,25,26]. Approaches to obtain good compaction density of the sulfur active material within the cathode composite are then covered in Section 2.2 [22,27,28]. Figure 2 provides a schematic outline of the topics to be covered in the entirety of Section 2.

### 2.1. Design of Cathode Composite Architecture

To increase sulfur loading and mitigate the effects of active material volume change in the cathode composite, nanosized sulfur is usually embedded in a porous host. This host is typically macroporous or mesoporous, providing a high surface area that allows for high loading of the active material as well as Li^+^/e^−^ conductive agents. Nanopores in the host material are also effective in anchoring soluble polysulfide species at the cathode side, helping to alleviate the effect of polysulfide shuttling and active material loss. Recent advancements have allowed for the fabrication of 1D, 2D, and 3D hierarchical conductive matrices for the encapsulation of sulfur to serve as cathode composites in large-scale Li–S pouch cells. Various electronically conductive carbon materials—such as carbon nanotubes (CNTs) [29,30], graphene [31,32], and carbon nanofibers (CNFs)—have been proposed as cost-effective host structures to combat the poor electronic conductivity of sulfur for scaling Li–S batteries to pouch cells with satisfactory cycling performance [33,34].

Carbon and CoS nanotubes with sufficient lumen space enable highly loaded sulfur cathodes with a highly electronically conductive network and robust architecture for efficient active material utilization [24]. The strong capillarity effect and chemisorption within the inner walls of the nanotubes immobilize the polysulfides and promote their redox kinetics. As a result, Li–S pouch cells utilizing this approach exhibited an initial capacity of 1330 mAh g^−1^ and an areal capacity of 5.05 mAh cm^−2^. Subsequent cycling showed the discharge capacity remaining steady at 1060 mAh g^−1^ (4.03 mAh cm^−2^) for 200 cycles under bending–unbending conditions, demonstrating the mechanical flexibility and stable cyclability of this scheme. Zhang et al. designed a 2D carbon host that consisted of graphitic carbon nanocages (GCNs) uniformly distributed on a graphene sheet backbone [35]. The graphitic nanoshells served as highly efficient electrochemical nanoreactors having excellent electronic conductivity and structural stability, which accelerate the kinetics of electron movement and prevent the diffusion of polysulfides, while the underlying graphene sheet served as the electronic pathway between each graphite shell. Pouch cells assembled with this cathode architecture and a sulfur loading of ≈77 wt% exhibited a capacity of ≈1 Ah. Hu et al. reported a hybrid graphene foam–reduced graphene oxide nested hierarchical network with nanosized sulfur composite cathode (GF–rGO/S) [26]. This composite contains a 3D interconnected framework of graphene foam which provides high electrical conductivity and a porous structure that has sufficient space to house sulfur active material, a large surface area, and a short Li^+^/e^−^ transfer pathway. Additionally, the rGO sheets of the GF–rGO/S composite provide anchoring sites for polysulfides that help prevent the shuttling effect. A pouch cell consisting of the GF–rGO/S cathode composite with a sulfur loading of 85.7 wt% (10 mg cm^−2^) and a cathode area of 10 cm^2^ delivered a specific capacity of 1235 mAh g^−1^ at a current density of 0.01 C.

These examples serve to illustrate that material/configuration design is a fruitful pathway to increase active material mass loading/utilization and suppress the detriments of active material volume change during cycling via simultaneously providing high-specific-areal space and regulating polysulfide shuttling in Li–S pouch cells. Moreover, a common theme among these examples is the structural stability of the cathode composite enabling high sulfur loadings in pouch cells operated under realistic conditions.

### 2.2. Cathode Compaction

In the past decade, carbon-based materials have been widely used in S cathodes, but various problems of carbon have also hindered the commercialization of lithium–sulfur batteries. Carbon-based material with a high specific surface area can serve as a superior host for sulfur, and it will also increase the interfacial resistance inside the electrode, and its lower mass density will seriously reduce the volumetric energy density of the battery [36]. Therefore, the carbon material must be in a reasonable specific surface area (porosity) range [20]. Besides, by tuning the morphology of the carbon host, the interfacial resistance can be reduced and the packed structure can be realized. However, carbon materials that do not contribute to the capacity will jeopardize the energy density of the pouch cell.

Pouch cells with high gravimetric and volumetric energy densities require active-material dense electrodes. This factor causes concern over porosity and the amount of non-redox-active agents in the sulfur cathode composites [20,37]. High-porosity conductive carbon additives and matrices in the cathode allow for high sulfur loading and effective electrochemical operation; however, they also cause additional electrolyte adsorption and the formation of more parasitic precipitates, which lead to cathode passivation and electrolyte depletion. Having a compact sulfur cathode is expected to restrain sulfur segregation, decrease electrolyte consumption, and reduce the mass-transfer resistance of polysulfide species in the cathode layer. Recent studies have shown that the compaction density of the cathode can be improved by adjusting the electrode compositions and employing external stacking pressure; the right-hand side of Figure 2 shows a schematic summary of these strategies.

Li et al. [22] introduced Chevrel-phase Mo_6_S_8_ into the cathode composite to form a hybrid intercalation–conversion cathode that effectively increases the tap density of the composite in pouch cells. The Mo_6_S_8_ offers an electronic matrix as good as carbon, greatly decreasing the necessary carbon content from ~30 wt% in traditional C/S_8_ cathodes to ~10 wt% to achieve the required electronic pathway in the cathode composite. Reducing the amount of carbon in the cathode composite for this system has the added benefit of decreasing the thickness of the electrode while maintaining its homogeneous morphology. The uniformly dispersed Mo_6_S_8_ allowed for the overall packing density of the cathode with a sulfur loading of 6.2 mg cm^−2^ to double and the amount of required electrolyte to be minimized owing to the reduced porosity of the electrode (from ~70% to 55 vol%). Ah-level pouch cells with this hybrid cathode and a low E/S ratio exhibited gravimetric and volumetric energy densities of 366 Wh kg^−1^ and 581 Wh L^−1^, respectively. The metal compound with high tap density, which can reduce volume variation and contribute capacity during charge and discharge, is a superior substrate for the conductive agent of next-generation Li–S batteries [22]. A metal compound with reasonable porosity, high tap density, excellent conductivity, and reversible capacity in the voltage range is the main basis for the design of the next generation of S cathodes. In detail, reasonable porosity and high tap density mean that the volume change can be alleviated, but the overall energy density is not reduced. Excellent electrical conductivity is an indispensable condition for metal compounds to replace carbon materials because free internal charge transfer is necessary for sulfur cathodes. The reversible capacity of some metal compounds can further increase the theoretical specific capacity of the sulfur cathode, which is not available in carbon materials.

Additional critical issues concerning the compaction of a cathode having a high sulfur content are the maintenance of adequate physical contact with the current collector during cycling and its resiliency to severe mechanical stress such as bending, rolling, folding, or the localized stress induced by the volume expansion of sulfur during cycling in a pouch cell. Alleviating these issues requires multifunctional binders to be added to the cathode composite. Conductive polymers—including aqueous and nonaqueous binders such as carboxymethyl cellulose (CMC), polyvinylidene fluoride (PVDF), polypyrrole (PPy), polyaniline (PANI), and poly(3,4-(ethylenedioxy)thiophene) (PEDOT)—have been widely used to mechanically stabilize the sulfur cathode from contact loss and aid in preventing polysulfide release [38]. A 3D-network electronically conductive binder with strong interchain interactions was introduced in a sulfur cathode by Liu et al. to ensure a mechanically robust electrode with a high sulfur loading [39]. The 3D-network binder has a high adhesive force and a high tolerance to volume change, allowing the use of cathode with high sulfur loading. A prototype cell having a cathode with 19.8 mg cm^−2^ sulfur delivered an areal capacity of 26.4 mAh cm^−2^ owing to the reduced modulus and hardness of the N–GG–XG binder. Moreover, this cell showed improved capacity retention, maintaining a capacity of 724 mAh g^−2^ after 150 cycles at 0.5 C, and outstanding rate capability, having a capacity as high as 737 mAh g^−1^ at a 5 C rate. These improved performance metrics are attributed to the effective binding of oxygen-containing functional groups in the biopolymer to the polysulfide species formed during cycling [39]. A water-based dual-cross-linked polymer binder (PACEC) was designed to maintain a compact electrode while aiding in preventing the shuttle effect of the cathode (Figure 2). The enhanced adhesion strength and remarkable LiS_x_ adsorption of PACEC allowed for a sulfur loading of 14.8 mg cm^−2^ in a crack-free cathode composite. Pouch cells with a cross-sectional electrode area of 6 cm^2^ and 177% lithium achieved a capacity of 6.5 mAh cm^−2^, with a Coulombic efficiency of 99.74% per cycle [27].

Volume expansion perpendicular to the face of the cathode in the pouch cells during cycling has been measured with a laser thickness gauge [40]. External pressure was used to achieve a highly compact cathode during cycling (Figure 2). Maiga et al. systematically studied the performance of Li–S pouch cells under various pressures ranging from 0 to 600 kg cm^−2^ [28]. The results showed that a pressurized cathode with 90 wt% sulfur loading was able to deliver an initial energy density of 11.7 mWh cm^−2^ and retain 8.4 mWh cm^−2^ after 900 cycles at a 0.2 C rate under 600 kg cm^−2^ of pressure. Even at a high areal sulfur-loading of 15.125 mg cm^−2^, an areal energy density of 19.24 mWh cm^−2^ was obtained. This value is 1.7 times larger than that of a commercial Li-ion battery. Using stack pressure during cycling offers a facile method to enable high-areal-capacity sulfur cathodes, which enhances the commercial viability of Li–S batteries. Table 2 summarizes effective cathode architectures for practical Li–S pouch cells. These strategies are still in their infancy and require additional development before they are industrially applicable. A cathode configuration must consist of at least 70 wt% sulfur to be commercially viable. Thus, the remaining weight of the cathode composite should consist of 10–15 wt% host material, 10–15 wt% conductive agent, and 3–5 wt% binder. These ranges provide a rough framework that can be adjusted based on the required application. In addition to a proper cathode architecture, operating pressure and current density should be given careful consideration for a Li–S pouch cell to obtain optimal electrochemical performance.

## 3. Electrolytes for Li–S Pouch Cells

The electrolyte not only provides ionic transport between the two electrodes in a Li–S battery but also serves as a reaction medium for the conversion of the sulfur species within the cathode. Electrolyte decomposition at the cathode and anode interfaces is the most prominent cause of performance degradation in Li–S pouch cells; it can lead to poor sulfur active material utilization, low rate capability, and a short cycle life [51]. In Li–S pouch cells with excess electrolyte (high E/S ratio), the cathode material is completely wet and the cell has a prolonged cycle-life because electrolyte depletion takes longer to occur [19]. However, a high E/S ratio leads to relatively low gravimetric and volumetric energy densities in cells; as the areal sulfur loading of a cell increases, the impact of the E/S ratio of the cell on the gravimetric energy density increases [20]. Thus, to enhance the specific capacity of Li–S pouch cells, it is of the utmost importance to improve their cyclability under lean electrolyte conditions (low E/S ratio).

**Figure 3:**
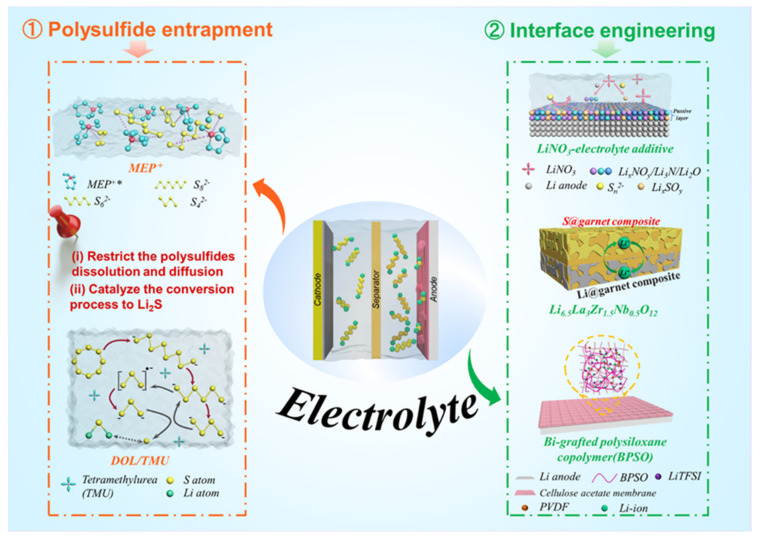
Strategies and perspectives to enable Li–S pouch cells under lean electrolyte conditions. (**1**) Regulate polysulfide species generated during the conversion process. (**2**) Engineering the interface between the electrolyte and the lithium metal anode.

Generally, Li–S pouch cells operating under lean electrolyte conditions face three main challenges: (1) the kinetics of cathode redox reactions are slowed and cause uneven wetting of the active materials, (2) the dissolution of polysulfide species causes severe electrolyte depletion, and (3) the electrochemically induced interfacial reactions with Li metal consume electrolyte to form a solid–electrolyte interphase (SEI) [19,52]. Additionally, the effect of the E/S ratio on the energy density of the cathode composite varies with sulfur loading and compaction density, even at E/S ratios as low as 3.0 µL mg^−1^ [53]. For large-scale Li–S pouch cells, the E/S ratio should be calculated and chosen based upon the cathode/anode composition and configuration. To enhance the kinetics of the electrochemical processes of the cell and reduce the effects of side reactions under lean electrolyte conditions, additives or novel electrolyte compositions are suggested to catalyze sulfur conversion, suppress the shuttle effect, and/or enable the formation of a stable passivation layer on the lithium metal anode (Figure 3) [53,54].

High-concentration electrolyte (HCE) systems can simultaneously inhibit the shuttle effect of lithium polysulfide and the growth of lithium dendrites. However, due to the use of a large amount of expensive Li salt, the cost of the electrolyte has always been high. In addition, the viscosity of the electrolyte will increase drastically, while the wettability will also decrease along with the high concentration of electrolyte. Accordingly, there will not be enough free solvent molecules to dissolve polysulfides, and the ion diffusion and reaction kinetics will be slowed [55]. This strategy does not seem to be the first choice for commercial Li–S batteries. Localized high-concentration electrolytes (LHCEs) show the advantages of high-concentration electrolytes and have the characteristics of low-concentration electrolytes with low viscosity and low cost [56]. Although LHCEs are promising candidates for the next generation of electrolytes, the ratio of various components in the electrolyte and the role of additives still require further research [57].

In addition, solid-state electrolytes are an alternative avenue of pursuit for Li–S pouch cells [58,59]. An all-solid-state Li–S pouch cell would negate any concern over polysulfide dissolution and shuttling. However, preliminary small-scale pouch cells with this approach have encountered dramatic problems [58]. Many of the solid-state electrolytes with sufficient ionic conductivity do not have an electrochemical stability window large enough to remain inert against lithium metal, causing sluggish Li^+^ transport across the lithium metal solid–electrolyte interface. The electrode–electrolyte interfaces also have poor mechanical integrality due to the nature of the solid–solid interface during charge/discharge cycling [17,60]. Attempts to accelerate the feasibility of solid-state electrolytes in Li–S pouch cells focus on interfacial engineering to avoid chemical and mechanical decomposition through such means as using thin-film solid-state electrolytes and softening interfacial contact during cycling (Figure 3) [61,62,63].

### 3.1. Strategies to Regulate Polysulfide Conversion under Lean Electrolytes

Uneven precipitation and accumulation of insulative sulfur/Li_2_S species can occur under lean electrolyte conditions, causing sluggish sulfur conversion in the cathode side. A high concentration of polysulfide species and a low electrolyte viscosity result in a charge carrier concentration gradient during cycling and cause a large voltage hysteresis [64,65]. This hysteresis limits the utilization of active material and the rate performance of the cell, making the mediation of polysulfide species in the electrolyte a greater challenge to overcome when a low E/S ratio is used. Electrolyte additives or organic solvents with a high donor number have been shown to increase the solvation of polysulfide species in the electrolyte and simultaneously trap high-order polysulfides to catalyze their conversion to Li_2_S, suppressing the shuttling effect.

Zhang and coworkers introduced a large size N-methyl-N-ethyl pyrrolidinium (MEP^+^) cation that binds and stabilizes polysulfide species in the electrolyte (Figure 3) [54]. From hard–soft acid–base theory (HSAB), MEP^+^ as a soft acid interacts strongly with soft bases—such as S_n_^2–^ species—which inhibits the disproportionation of polysulfides in the electrolyte. This addition of MEP^+^ to the electrolyte enabled a 5 Ah pouch-type LSB that showed a high initial energy density of over 300 Wh kg^−1^ and a cycle-life of 100 cycles. High-dielectric solvents are expected to enhance the solvation of sulfur species and improve sulfur utilization in the cathode. Modified ether electrolytes based on a high-ε aprotic solvent of tetramethylurea (TMU) (Figure 3) have been proposed to regulate the viscosity and polysulfide conversion in cells with a low E/S ratio [53]. Combined with DOL, a DOL/TMU electrolyte can efficiently solvate S_3_^•–^ radicals, accelerate polysulfide conversion, and have good anodic stability against Li metal. A pouch cell using a TMU-based high-ε electrolyte delivered a sulfur utilization of 91% and a high energy density of 324 Wh kg^−1^ with an E/S ratio of 3 μL mg^−1^. Kaskel et al. combined tetramethylene sulfone (TMS) with a solvent blend of 1,1,2,2-tetrafluoroethyl-2,2,3,3-tetrafluoropropyl ether (TTE) to mediate polysulfide solubility within the cell [66]. This system (LiTFSI in TMS/TTE) had a reduced polysulfide concentration gradient during cycling and achieved a high Coulombic efficiency above 94% with an E/S ratio below 2.6 μL mg^−1^, offering a promising route toward practical high-energy Li–S pouch cells.

Li–S pouch cells with a high energy density can be obtained with a low E/S ratio by mediating the solvation of sulfur species and the conversion kinetics via the electrolyte. The compromise between high sulfur utilization and a low E/S ratio is also associated with the composition and compaction density of the cathode composite. Thus, although the electrolyte must be given careful consideration for optimum large-scale Li–S pouch cell performance, maximizing the cell’s energy density with a high sulfur loading and low E/S ratio needs further elucidation before it becomes a practically viable approach.

### 3.2. Interfacial Engineering

Li–S pouch cells with a high sulfur loading and a low E/S ratio can have a higher concentration of polysulfides within the electrolyte. This effect leads to more pronounced shuttling and severe chemomechanical reactions at the electrolyte–electrode interfaces that can form an unstable SEI [65]. An unstable SEI layer at the electrode–electrolyte interface allows polysulfides to penetrate the interior of the Li anode and form new SEI and “dead Li”, further depleting both the electrolyte and the lithium metal anode during extended cycling. Moreover, the thick SEI layer blocks Li^+^ and electron transfer to the lithium anode and can cause a localized current density higher than the critical current density for forming lithium dendrites. This high localized current density triggers the onset of lithium dendrite formation, which can ultimately short-circuit the cell. Interfacial engineering is essential in preventing these catastrophic outcomes in the practical operation of pouch cells with high sulfur loading under lean electrolyte conditions. The main electrolyte-tailoring strategies used to improve the performance of the anode–electrolyte interface in Li–S pouch cells are (1) in situ formation of a reinforced passivation layer on the Li anode surface via an electrolyte additive, (2) using a solid-state electrolyte to block polysulfide shuttling and suppress lithium dendrite growth, and (3) employing a polymer electrolyte with limited polysulfide species solubility and high stability against a Li metal anode.

#### 3.2.1. Additives in Liquid Electrolyte for SEI Construction

Li–S cells typically employ ether-based electrolytes, in which lithium salts (LiTFSI) are dissolved in a combination of 1,3-dioxolane (DOL) and 1,2-dimethoxyethane (DME) solvents [67]. Lithium nitride (LiNO_3_) is a widely adopted additive for these liquid electrolytes (Figure 3) owing to its ability to effectively suppress the decomposition of the electrolytes and induce the polymerization of DOL to form a polymeric layer to passivate the lithium metal anode [68]. LiNO_3_ also aids in hindering the negative effects of dissolved polysulfide species on the Li metal anode by oxidizing the polysulfides to Li_x_SO_y_. SEI layers formed in the presence of LiNO_3_ have been shown to have a more optimal composition for Li–S pouch cell performance. However, the exact composition of this SEI is yet to be identified, it is known to be a complex mixture of organic (e.g., ROLi, ROCOLi, RCOOLi, where R is the organic group) and inorganic compounds (e.g., LiF, Li_2_O, Li_3_N, Li_2_S, LiS_x_O_y_, Li_x_NO_y_) [50,61,69,70]. Table 3 presents a performance comparison of Li–S pouch cells with and without LiNO_3_ additives in their electrolytes using various cathode architectures. The SEI layer formed with a LiNO_3_ additive effectively suppresses parasitic reactions of the polysulfides with lithium metal to prevent the formation of Li dendrites in the Li–S pouch cells during operation. Long-term galvanostatic cycling tests showed that pouch cells with a LiNO_3_ electrolyte additive maintained a stable capacity for up to 100 cycles with a sulfur loading of 6 mg cm^−2^ [71]. Other functional additives with a high donor number, such as salts containing the NO_3_^−^ anion, have also been explored in Li–S cells to suppress electrolyte decomposition on the Li metal by regulating the Li^+^ solvation shell. This regulation enhances the cycling performance of the lean electrolyte Li–S pouch cells. Fluorinated solvents with lower viscosity can form a LiF-rich SEI on the lithium metal. LiF is regarded as a good SEI component for stabilizing the lithium metal surface and allowing Li-ion transfer while suppressing dendrite nucleation. This enhanced LiF-rich SEI is effective in yielding a safe and practical Li–S cell with improved cycle life [72].

However, these functional additives are constantly consumed at both the anode and the cathode during cycling and cannot maintain a long cycle-life at the pouch cell level. Thus, exploring new electrolyte additives that can decrease parasitic side reactions and enhance the stability of the SEI layer in Li–S cells is a worthwhile endeavor for realizing industrially relevant Li–S pouch cells.

#### 3.2.2. Inorganic Solid-State Electrolytes

Inorganic ceramic solid electrolytes do not solvate polysulfide species and are chemically/electrochemically stable with sulfide species, making them a prime candidate for Li–S cells with a high sulfur utilization. As shown in Figure 3, a porous–dense–porous trilayer garnet-type solid-state electrolyte was designed for a Li–S cell to block polysulfide shuttling and prevent lithium dendrite growth; the thick porous layer acts as a robust mechanical support and a sulfur host [62]. A 5 cm × 5 cm pouch cell with this trilayer garnet-type solid-state electrolyte showed a high energy density and stable cycling.

Sulfide-based solid electrolytes are easy to process, have high ionic conductivity, and have the lowest electronic conductivity at room temperature [75]. These characteristics allow them to be integrated into Li–S cells with limited volume and a soft interface contact. There are numerous techniques for obtaining optimal interfaces for Li^+^ transfer. Hot pressing, slurry coating, and the use of interfacial buffer layers are among the most popular methods for obtaining good interfacial compatibility in all-solid-state Li–S pouch cells with sulfide solid electrolytes [58,76]. Large-scale all-solid-state Li–S pouch cells with a sulfur cathode and Li_10_GeP_2_S_12_ electrolyte yield a high specific capacity of 1169 mAh g^−1^ and excellent cycling stability [77]. Although the sulfide solid electrolyte possesses high Li^+^ conductivity, its development is still hindered by many of its characteristics [58]. Narrow electrochemical window (1.5~2.5 V) and unstable electrode–electrolyte interface affect its long-term cycling performance.

Although the suppression of polysulfide shuttling and lithium dendrite growth in Li–S cells can be accomplished with solid-state electrolytes, there are still challenges in forming thin and dense electrolytes that maintain their mechanical integrity during cell packaging. This critical factor strongly hinders the practical feasibility of all-solid-state Li–S pouch cells.

#### 3.2.3. Polymer-Based Solid Electrolytes

Polymer-based solid electrolytes are generally easy to process and have outstanding flexibility at room temperature [78]. Li–S pouch cells with a polymer-based electrolyte have been demonstrated to have negligible polysulfide shuttling while maintaining good interfacial contact with both electrodes. For polymer-based solid electrolytes, improving Li^+^ conductivity is of great significance for short-term cycling [79]. As for the long-term cycling of polymer-based solid electrolytes, determining how to suppress the growth of lithium dendrites is the first key point; for example, it could be improved by increasing mechanical strength [58]. Armand et al. introduced LiDFTFSI–PEO as a polymer electrolyte for LSBs [80]. In this system, a CF_2_H moiety is beneficial in strengthening the interaction between DFTFSI^−^ and poly(ethylene oxide) (PEO). LiDFTFSI not only inhibits polysulfide shuttling but also induces the formation of a robust SEI on the lithium anode surface. Consequently, LiDFTFSI-based LSBs showed a high areal capacity and gravimetric energy density. Tao et al. integrated a ceramic Li-ion conductor (garnet-type Li_7_La_3_Zr_2_O_12_ (LLZO)) into PEO to form a composite solid-state electrolyte for all-solid-state Li–S cells that operate well at 37 °C [81]. Li–S pouch cells with a S@LLZO@C composite cathode and the PEO–LLZO composite electrolyte reached a specific capacity of 900 mAh g^−1^ and retained a capacity of 800 mAh g^−1^ after 200 cycles.

PVDF has a low donor number, which is proposed to restrain the formation of soluble polysulfides and manipulate the conversion of sulfur from a multistep “solid–liquid–solid” process to a single-step “solid–solid” reaction, making the PVDF electrolyte a competitive polymer electrolyte for Li–S cells (Figure 3) [59]. Fan et al. reported a composite polymer electrolyte (CPE) consisting of PVDF, bi-grafted polysiloxane copolymer (BPSO), and cellulose acetate (CA) [63]. This membrane delivers an ionic conductivity of 7 × 10^−4^ S cm^−1^ at 25 °C and forms a stable interface with a lithium anode, working well in an MCNT@S/90% (BPSO–150% LiTFSI)–10% PVDF^+^ CA/Li pouch cell.

The electrolyte is the most critical component of combating polysulfide shuttling and interfacial instability with the lithium metal anode in Li–S pouch cells. Bringing the E/S ratio to a commercially feasible value while circumventing the aforementioned issues only adds to the challenge. Additives and SEI reformation have been adopted to stabilize polysulfide species, decrease interfacial side reactions, and inhibit the nucleation of lithium dendrites in Li–S pouch cells. Thus, additives and progressive alternative electrolyte strategies are a promising route for discovering an optimal electrolyte composition that has moderate to low polysulfide solubility and good compatibility with lithium metal for fabricating competitive Li–S pouch cells.

## 4. Separator Modification

Traditional polyolefin separators—such as polypropylene (PP) and polyethylene (PE)—have been widely used in Li–S cells due to their low price, mechanical strength, thermal stability, and electrolyte wettability. Separators in Li–S cells are responsible for sieving ions, mediating polysulfide shuttling, and blocking cell shorting from Li dendrite growth. However, soluble polysulfide species generated during sulfur conversion in the cathode side can dissolve in the electrolyte and pass through the separator to the anode side. Once at the anode side, the polysulfide species induce parasitic reactions that poison the surface of the anode, causing severe capacity attenuation. Moreover, PP and PE separators have low thermal conductivity (<0.40 W m^−1^ K^−1^) and thermal diffusivity (<2.60 mm^2^ s^−1^), making it difficult to dissipate the heat generated inside the battery, increasing the risk of thermal runaway in Li–S pouch cells [82].

**Figure 4:**
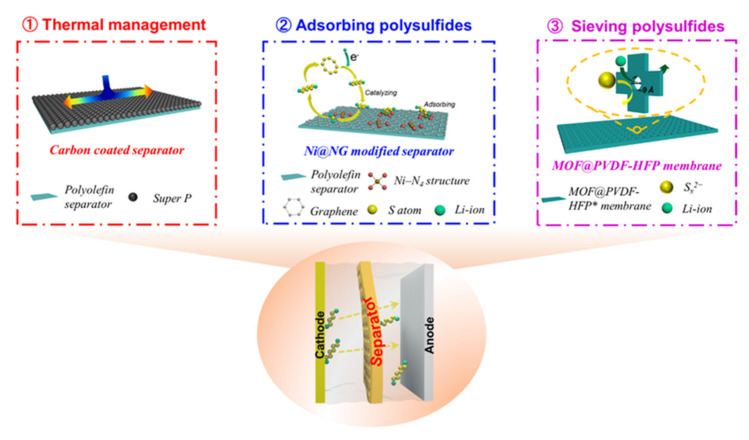
Separator strategies to enable Li–S pouch cells. (**1**) Thermal management coatings and (**2**) polysulfide restriction are the two main categories being pursued for tailoring Li–S pouch cell separators.

Given the dissolution of polysulfide species in the electrolyte, the ability of a separator to trap polysulfide species is crucial for efficient cell performance. Appropriate thermal management of the separators may provide additional safety and stability of the pouch cell by limiting thermal runaway. Carbon-based materials have relatively high thermal conductivity, which helps separators to rapidly remove heat under heat-producing reactions (charge/discharge process, interfacial kinetics, or an internal short circuit condition). The shrinkage of PP/PE separators under heat can be greatly reduced through surface modification with a carbon additive [82]. Functional separators have been developed to ameliorate heat accumulation and restrict polysulfide permeation. Figure 4 schematically illustrates strategies being pursued to beneficially alter separators for Li–S battery operation. Efforts to improve the properties of separators in Li–S pouch cells can be divided into two main categories: (1) thermal management [82] and (2) polysulfide restriction [74,83].

### 4.1. Thermal Management of Separators

Poor thermal management causes pressure accumulation through internal component reactions, which serves as a safety risk with the potential for thermal runaway of the battery. Among various possible heat dissipation interlayers, inorganic oxide and lightweight carbonaceous materials are some of the most cost-effective options to enhance the thermal conductivity of PP/PE separators (Figure 4). Carbon black [82], CNTs [84], and Al_2_O_3_ have been proposed to functionally regulate the thermal dissipation of separators in Li–S pouch cells [85]. Meng et al. reported a carbon black-coated PE separator (LA132–C) with an extremely high in-plane thermal diffusivity of 32.47 mm^2^ s^−1^ and in-plane thermal conductivity of 8.43 W m^−1^ K^−1^ (228% of that of commercial separators) [82]. This LA132–C separator possesses a puncture strength of 10.8 MPa, which is higher than plain PP (3.60 MPa) or plain PE (8.53 MPa) separators. During a pouch cell abuse test, the heat produced inside the cell could quickly diffuse through both the in-plane and cross-plane directions of the LA132–C separator. An IR camera was used to verify the temperature distribution of a cell with a LA132–C separator under an external short circuit (ESC) test. The temperature was more homogeneous for the cell with the LA132–C separator than for the cells with PE and PP separators. Li–S pouch cells with the LA132–C separator and an E/S of 4.3 achieved a first discharge specific capacity of 1383.7 mA h g^−1^ at a 0.01 C rate and retained a capacity of over 87.7% after 30 cycles, which is much higher than the cell with a PE separator which only retained 46.2% of its initial discharge capacity after 30 cycles at a 0.05 C rate.

Heat dissipation is an important aspect of cell design and can be accounted for through engineering the separator of a pouch cell. Separator coatings give means to improve the thermal conductivity and mechanical properties of polymer separators, which greatly reduce heat accumulation inside the cell and decrease the risk of thermal runaway during operation. In some degradation studies, the contributions of cell degradation depend upon cell component chemistry, but in pouch cells, the thermal runaway should be considered if the capacity degradation appears when the cell temperature increases.

### 4.2. Polysulfide Restriction

Although lightweight nanocarbon separator coatings have shown the ability to anchor polysulfides through physical interactions to some extent, they leave much to be desired in preventing polysulfide shuttling. Other compounds such as metal oxides [14,85,86], sulfides [87,88], and phosphides have shown promise to exceed the performance of carbonaceous materials as a coating on traditional PP/PE separators to block polysulfide shuttling [89]. Manthiram et al. synthesized vertical Co_9_S_8_ hollow nanowall arrays in situ on a Celgard membrane with a loading of only 0.16 mg cm^−2^ [88]. The polar Co_9_S_8_ hollow array firmly anchored polysulfide species at the cathode side through chemical and physical adsorption, thereby effectively restricting the shuttle effect. A pouch cell with a sulfur loading of 2 mg cm^−2^ and a Co_9_S_8_-coated PP separator exhibited a specific capacity of 1185 mAh g^−1^ and remained stable for 30 cycles.

Studies have shown that single atoms with 100% reaction efficiency can catalyze the reaction kinetics of intermediates in a Li–S battery. Niu et al. prepared a nitrogen-doped graphene with an Ni–N_4_ structure (Ni@NG) on the separator [83]. In this scheme, the Ni atoms acted as active sites for adsorbing polysulfides (Figure 4) to form S_x_^2−^ Ni–N bonds. Meanwhile, charge transfer between the oxidized Ni atoms and the polysulfide species accelerated the redox conversion of polysulfides, improving the utilization of active sulfur species. A pouch cell assembled with this Ni@NG separator exhibited a high initial capacity of 1301.0 mAh g^−1^ at 0.1 C rate and 580.8 mAh g^−1^ at 3.0 C rate. After 200 cycles at a 1 C rate, the cell retained 80.5% of its capacity.

Aside from PP/PE separator coatings, metal–organic frameworks (MOFs) with intramolecular pores are unique materials that can act as molecular sieves to selectively block polysulfide migration from the cathode to the anode in a Li–S cell (Figure 4). A flexible MOF@PVDF–HFP separator proposed by Zhou et al. showed improved mechanical properties and the ability to block polysulfide migration [74]. Pouch-type Li–S cells with a sulfur loading of 5.8 mg cm^−2^ were assembled with this MOF@PVDF–HFP separator; they delivered an initial capacity of 1269 mAh g^−1^ and retained a capacity of 936 mAh g^−1^ after 200 cycles.

Table 4 summarizes the performance of Li–S pouch cells with the aforementioned separator strategies. Casting a functional coating on a traditional polyolefin separator or using an MOF-based separator are the two most promising strategies for improving the safety of Li–S pouch cells while enhancing their electrochemical performance and thermal characteristics. Carbon-based polyolefin separator coatings provide a high thermal conductivity and facilitate fast heat dissipation in the cell. MOF-based separators are exceptional at sieving polysulfide anions to prevent their migration to the anode. Each of these approaches also provides the added benefit of improving the mechanical properties of the separator, which aids in preventing lithium dendrite growth. These findings have sparked investigation into separator engineering specifically for Li–S pouch cells and its effect on cell safety. Further research into each of these aspects of separator design is necessary before they can be applied commercially.

## 5. Strategies for the Lithium Metal Anode

**Figure 5:**
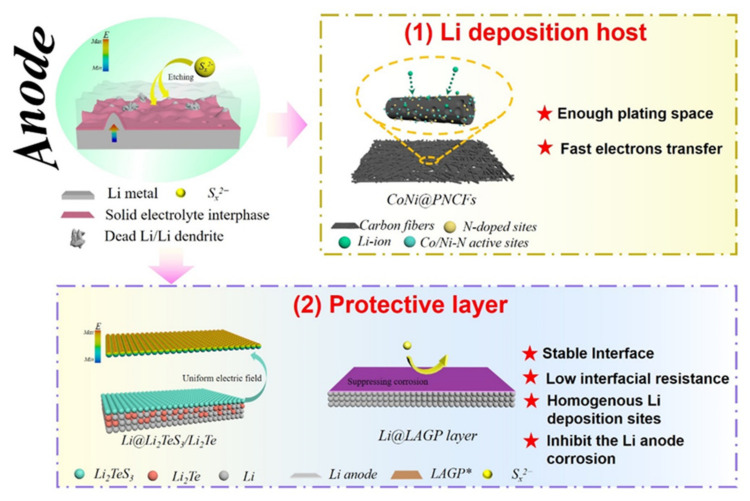
Approaches for stabilizing the lithium metal anode in Li–S pouch cells. (**1**) Nanostructured lithium deposition host and (**2**) artificial protective layers are the two dominant strategies being pursued for overcoming lithium volume change, dendrite growth, SEI cracking, and corrosion of the lithium metal anode.

Polysulfide shuttling is the main cause of failure in coin cells. However, the lithium metal anode is the primary cause of failure in pouch cells [21,90]. At large current densities, the deposition of mossy Li and volume expansion during plating–stripping damage the interface contact, which induces greater cell polarization. These factors ultimately deteriorate the performance of the pouch cell during extended cycling. It is common to use a large excess of lithium in a Li–S battery. However, the formation and shuttling of polysulfide species can cause the formation of a thick SEI and severe lithium anode corrosion. Upon expansion and contraction of the lithium metal anode during cycling, the SEI layer is easily damaged and results in the formation of a new SEI where fresh lithium metal is exposed by the cracks. SEI phases are intrinsically electronic insulators. This property often leads to the deposition of “dead Li”. The loss of active lithium metal from the anode and the high resistance of the SEI layer contributes to the low Coulombic efficiency and poor cycling performance of large-scale Li–S cells. Current strategies aim to homogenize Li^+^ deposition, reduce “dead Li”, and restrain the parasitic corrosion of the lithium metal anode by using lithium deposition hosts and artificial protective layers on the surface of the anode (Figure 5) [15,73,91].

### 5.1. Hosts for Metallic Lithium Deposition

Localized electric fields and concentration polarizations are the primary cause of uneven lithium deposition and lithium dendrite formation at the anode side [92]. Therefore, homogenizing the electric field at the electrolyte–anode interface is crucial to enable uniform Li^+^ ion deposition during cycling. Host structures are an ideal way to accomplish this task while having the added benefit of being able to mitigate metallic lithium volume expansion and tailor lithium nucleation sites within the host architecture [93]. Regulating these two factors is an effective way to inhibit the growth of mossy Li in Li–S cells (Figure 5). Lightweight nanomaterials that are porous enough to serve as a host for plated lithium metal and provide fast electron transfer are highly recommended for stabilizing the lithium metal anode in Li–S pouch cells.

For instance, flexible carbonaceous materials with a high specific surface area fit the requirements of an efficient lithium metal host. The porosity and high electronic conductivity provide a large deposition surface and uniform lithium nucleation sites. He et al. proposed a flexible CoNi nanoparticle-embedded porous conductive scaffold (CoNi@PNCFs) as a deposition host for a Li–S pouch cell anode [73]. The porous scaffold and CoNi nanoparticles in CoNi@PNCFs provide abundant nucleation sites and a uniform electric field to yield a homogeneous local current density for Li plating (Figure 5). Luo et al. designed a mechanically robust r-GO-based anode for Li–S pouch cells that is tolerant to extreme bending. The r-GO served as a support for lithium metal deposition and had a more evenly distributed current density during lithium plating–stripping [94]. As a result, mossy Li deposition and corrosion due to polysulfide species were halted. The Li–S pouch cell using the rGO/Li anode delivered a capacity around 900 mAh g^−1^ and maintained stable operation for over 100 cycles at a current density of 0.1 A g^−1^ while the cell was bent.

Providing a flexible host matrix for a lithium anode is a promising strategy for achieving structural stability while accommodating the volume change incurred during Li stripping–plating. A host with a high active surface area and nucleation sites of equal energy can help to unify the local electric field and decrease the probability of Li dendrite growth at the anode.

### 5.2. Artificial Protective Layer

When mossy lithium and dendritic lithium form, they are quickly oxidized in the presence of an organic electrolyte and dissolved polysulfides. This process, which generates new SEI on the exposed lithium metal anode, continues indefinitely to deplete the electrolyte and results in a thick interphase layer that enables the formation of “dead Li”. This issue becomes more severe in Li–S cells with high sulfur loading under lean electrolyte conditions where the high-concentration polysulfide species add an additional contribution to the SEI layer formation by shuttling to the anode and corroding the lithium metal [9]. A mechanically robust protection layer on the lithium metal surface is a proposed strategy to alleviate these issues. Various inorganic and organic materials (such as Al_2_O_3_, Li_3_PO_4_, Li_3_N, LiPON, polymer) have been deposited on the surface of lithium metal to form a high-quality artificial protective layer in Li metal batteries. Herein, facile and economical approaches to this strategy are discussed for Li–S pouch cells with a high sulfur loading and low E/S ratio.

For example, an ultrathin layer of Mg deposited on Li metal can form a Li-rich alloy that has a low charge transfer energy barrier and only a moderate surface reactivity during cycling [95]. Lithium metal is able to uniformly plate–strip through this Li_x_Mg layer, which has a morphology that remains stable under repeated cycling. This thin alloy layer also inhibits the side reactions between the anode and electrolyte components, especially polysulfide species. Researchers have also developed in situ electrochemical deposition strategies to form stable artificial interphase layers on a lithium metal anode. Manthiram et al. stabilized Li deposition by introducing tellurium (Te) as an additive in a cell with limited lithium in the anode and a low E/S ratio [91]. The in situ formed tellurium and sulfide-rich SEI film on the lithium surface significantly improved the reversibility of Li plating–stripping. Specifically, during the lithiation process, Te can form a novel bilayer SEI consisting of Li_2_TeS_3_ and Li_2_Te on the anode surface (Figure 5). This SEI layer provides a lower Li^+^ diffusion barrier energy that helps the homogeneous deposition of lithium. Interfacial and charge-transfer resistances are also lowered with this strategy, which mitigates electrolyte decomposition and enhances interfacial stability at the anode. A large-area pouch cell (39 cm^−2^) with this Te additive operating under lean electrolyte conditions (4.5 μL mg^−1^) and a cathode sulfur loading of 5.2 mg cm^−2^ showed enhanced cycling stability.

Artificial protective layers have also been used to control the mechanical integrity of the anode–electrolyte interface. Some of the fabricated artificial layers have successfully enabled intimate contact with lithium metal while remaining chemically compatible with the reactive Li metal anode. Wen et al. proposed a composite protective layer (CPL) with LAGP (Li_1.5_Al_0.5_Ge_1.5_(PO_4_)_3_) for this purpose [15]. The LAGP CPL effectively inhibits the lithium metal corrosion from polysulfides and also suppresses the growth of lithium dendrites owing to its high Young’s modulus (Figure 5). A pouch cell with this LAGP CPL retained ∼74% of its initial capacity after 50 cycles at a 0.5 C rate. A protective layer consisting of Li_3_PS_4_ and its derivatives was developed by Wen et al. [16]. The facile Li^+^ transfer and soft–glassy nature of Li_3_PS_4_ made the artificial SEI layer able to mitigate the negative effects of anode volume change during cycling and enhance the interfacial stability during Li^+^ plating. A pouch cell with a sulfur loading of 3.8~4.2 g cm^−2^ and a LiNO_3_-free electrolyte delivered a discharge capacity of 803 mAh g^−1^ after 20 cycles and a stable Coulombic efficiency.

The use of lithium metal anodes for soft-pack batteries faces a very difficult challenge. For uniform and sustainable lithium plating–stripping over extended cycling, there must be uniform lithium nucleation sites and uniform electric field strength, and the generation of mossy or dendritic lithium must be suppressed. These requirements can be met through specific strategies for tailoring the anode structure and surface, such as using a host structure that can house metallic lithium within its pore space or through the construction of a stable artificial interfacial layer on the surface of the lithium metal anode that promotes uniform lithium plating–stripping and inhibits the occurrence of parasitic side reactions. Each of these approaches can effectively enable a lithium metal anode in a small-sized pouch cell. It is worth noting that the stability of lithium anodes differs greatly between small-current coin cell-type batteries and high-current pouch cells. One must pay close attention to the behavior of lithium anodes under various current densities in large-sized pouch cells.

## 6. Safety Concerns

The safety of pouch cells is a comprehensive problem, and the main obstacles come from Li dendrites and thermal management. The uneven electric field distribution and the sluggish Li^+^ diffusion rate cause the extreme growth of lithium dendrites in the vertical direction and the short circuit [2]. The problem of Li dendrites can be solved in the following ways: (1) accelerating the diffusion rate of lithium ions on the anode and inhibiting lithium dendrites, including the construction of robust artificial SEI and the modification of lithiophilic materials; (2) using lithiophilic and conductive materials to homogenize the electric field strength and promote the uniform plating of lithium metal; and (3) improving the mechanical strength of the separator and resisting the puncture of Li dendrites. As for the safety issues caused by thermal runaway, separator modification is believed to be able to effectively alleviate thermal runaway [82]. Because PP and PE have poor thermal conductivity, they lead to heat accumulation, which is especially obvious in the presence of dendrites. The development of a new type of membrane with better fireproofing also has a bright future, because it can avoid the use of PP and PE membranes [96]. In addition, replacing the electrolyte system or adding thermally stable additives to the electrolyte can also avoid safety problems caused by thermal runaway; for example, electrolytes with high boiling points and high flash points can be used as replacements, or flame retardants can be added [56]. At present, there is little research in this direction for Li–S batteries.

## 7. Conclusions and Perspectives

The main challenges of Li–S pouch cells have been outlined, and promising strategies for overcoming each of these issues have been summarized. To obtain a Li–S pouch cell with efficient electrochemical performance and a long cycle-life, the polysulfide shuttling effect and the problems related to the lithium metal anode must be addressed. Additionally, there is still work to be done on the cathode side in terms of active material utilization and sulfur loading before industrially relevant gravimetric and volumetric energy densities can be realized. Well-designed structures for cathode and anode materials can provide stable hosts for sulfur active material and lithium metal deposition, respectively. The design of matrix architectures for the sulfur cathode and lithium metal anode are still in their initial stages. To further this field, the needs to be a unified approach to assessing the commercial relevance of such cell design parameters as cathode densification, host swelling with electrolyte injection, thermal simulation, and stack pressure. A protective artificial interfacial layer on the lithium metal anode surface can effectively suppress lithium dendrite nucleation, inhibit parasitic side reactions, and limit the need for excess lithium metal and electrolyte. This crucial feature of this approach directly increases the energy density of Li–S pouch cells by reducing the weight and volume of components in the cell. Key properties for an ex situ or/and in situ prepared SEI, including composition, mechanical strength, (electro)chemical properties, density, thickness, and electronic/ionic conductivity, are not systematic and should be assessed and characterized in a cell with a low E/S ratio. Further, our scientific understanding of their role and their commercial feasibility in large-scale Li–S pouch cells should be noted. In addition, the investigation of electrolytes should be carefully designed with careful attention given to such parameters as ionic conductivity, electrolyte uptake, and the compatibility of the cathode/anode with thermal safety improvement. Adding functional additives or employing novel liquid- or solid-state electrolytes that can enable lean electrolyte conditions while having a large electrochemical window and stable interfacial compatibility would be a significant step forward in enabling practical Li–S pouch cells. Separators are of note for Li–S batteries because they can directly restrict the shuttling of polysulfide species. Their porosity, mechanical and thermal properties, electrolyte uptake, and volume expansion rate with electrolyte should not be neglected when assessing the safety factors of Li–S pouch cells.

The realization of large-scale Li–S pouch cells with a high energy density and a long cycle-life is a complex topic with many factors and mechanisms to contemplate. Sulfur utilization, cathode densification, electrolyte volume, lithium amount, interfacial design, electrode architecture, external stack pressure, operating parameters, and safety need to be carefully considered based on the desired commercial application for such cells. Future work on solving these issues is a key step towards reaching commercial Li–S pouch cells for a sustainable future.

## Figures and Tables

**Table 1:** Current status of Li–S coin cell and pouch cell electrochemical performances.

Coin-Type Cell ^1^					Pouch-Type Cell ^1^					
Areal Sulfur Loading (mg/cm^2^)	Diameter (mm)	Current Density	Specific Capacity (mAh g^−1^)	Cycles/Final Capacity (mAh g^−1^)	Areal Sulfur Loading (mg cm^−2^)	Area of Cathode (mm^2^)	Current Density	Specific Capacity (mAh g^−1^)	Cycles/Final Capacity (mAh g^−1^)	Ref.
–	–	0.2 C	1130	100/907	4	77 × 50	0.1 C	995	100/891	[13]
0.75	–	0.2 C	1067.7	100/804.4	2.4	40 × 30	0.1 C	957.7	20/780.9	[14]
~1.3	12	0.5 C	1154	100/832.1	~1.3	54 × 72	0.5 C	~1000	50/~750	[15]
~1.7	12	0.3 C	~1150	200/840	~4.0	–	0.2	~1200	20/~800	[16]

^1^ The related parameters are based on the S/Ketjenblack cathode.

**Table 2:** Summary of Li–S pouch cells ^1^ fabricated with different cathode architectures.

Cathode Configuration	Areal Sulfur Loading (mg cm^−2^)	Electrolyte	C Rate	Specific Capacity (mAh g^−1^)	Ref.
Oxidized TiN–ordered mesoporous carbon/S	~1.4	DOL/DME	0.1	884	[41]
Carbon cloth@carbon-encapsulated CoP nanosheet arrays/S	3.4	DOL/DGM	0.1	1100	[42]
CoS_2_–sulfurized polyacrylonitrile–CNT	5.9	EC/DMC/DEC	0.2	~1600	[43]
S@CNTs/CoS nanostraws	3.8	DOL/DME	0.1	1330	[24]
CNT@nitrogen-enriched carbon/S	4	TEGDME	–	1300	[44]
N-doped Ketjenblack/S	2.4	DOL/DME	–	~900	[45]
Graphene foam–rGO/S	10	DOL/DME	0.04	1235	[26]
Polar hierarchical–porous carbon container@S	4	DOL/DME	0.2	733	[46]
S/(graphene–graphitic carbon nanocages)	3	DOL/DME	0.05	~1000	[35]
S/conductive carbon	4.5	DOL/DME	0.05	1490	[47]
S/Ketjenblack–Ni–P layers	2	DOL/DME	0.1	1420	[48]
Pure sulfur cathode	~10.4	DOL/DME	0.05	1160	[49]
Sulfurized carbonized polyacrylonitrile	3	EC/DEC	0.5	~1300	[50]

^1^ The anode of pouch cells in Table 2 is metallic lithium.

**Table 3:** Electrochemical performance of Li–S pouch cells with liquid electrolytes.

Cathode	Areal Sulfur Loading (mg cm^−2^)	Electrolyte	Additive	Anode	C Rate	Specific Capacity (mAh g^−1^)	Ref.
S/Ketjenblack	~1.3	DOL/DME	–	LAGP/Li metal	0.5	~1000	[15]
S/Ketjenblack	~4.0	DOL/DME	–	Li_3_PS_4_/Li	0.2	~1200	[16]
S/CNTs	2.5	DOL/TMU	0.3 M LiNO_3_	Li metal	0.05	1524	[53]
S/Super C65	~3.9	DOL/DME	0.4 M LiNO_3_	Li metal	0.2	1205	[2]
S/CNTs	7.8	DOL/DME	1 wt% LiNO_3_	Li metal	0.1	1135	[71]
S/CoNi@PNCFs	~1.5	DOL/DME	0.1 M LiNO_3_	Li/CoNi@PNCFs	0.2	~1200	[73]
RGO@S	5.8	DOL/DME	0.2 wt% LiNO_3_	Li metal	0.1	1269	[74]

**Table 4:** Performance Li–S pouch cells ^1^ with novel separators.

Cathode	Areal Sulfur Loading (mg cm^−2^)	Electrolyte	Additive	E/S Ratio (µL/mg)	Separator	C Rate	Specific Capacity (mAh g^−1^)	Ref.
S/Ketjenblack	3	DOL/DME	–	4.3	Carbon/PE	0.01	1383.7	[82]
S/Ketjenblack	2.4	DOL/DME	0.1 M LiNO_3_	–	Graphene /PP/Al_2_O_3_	0.1	957.7	[14]
S/Super C65	~3.9	DOL/DME	0.4 M LiNO_3_	~3.3	PP@Mo_6_S_8_	0.2	1205	[87]
S/Super–P	2	DOL/DME	0.1 M LiNO_3_	10	Co_9_S_8_/PP	–	1185	[88]
S/CNTs	7.8	DOL/DME	1 wt% LiNO_3_	2 mL ^2^	PG/PP	0.1	1135	[71]
S/CNTs–acetylene black	4.6	DOL/DME	0.4 M LiNO_3_	1.5 mL ^2^	MoP/rGO/PP	0.1	1083	[89]
RGO@S	5.8	DOL/DME	0.2 wt% LiNO_3_	–	MOF@PVDF–HFP	0.1	1269	[74]

^1^ The anode of pouch cells in Table 4 is metallic lithium. ^2^ The dosage is based on the whole pouch cell.

## Data Availability

Not applicable.
